# Endoscopic Ultrasound Elastography in the Assessment of Rectal Tumors: How Well Does It Work in Clinical Practice?

**DOI:** 10.3390/diagnostics11071180

**Published:** 2021-06-29

**Authors:** Adrian Catinean, Gheorghe G. Balan, Anita Mezei, Emil-Claudiu Botan, Andrei-Otto Mitre, Radu Motocu, Florin Graur, Dan-Tudor Eniu, Maria-Adriana Neag

**Affiliations:** 1 Department of Gastroenterology, Emergency Clinical County Hospital, Iuliu Hatieganu University of Medicine and Pharmacy, 400006 Cluj-Napoca, Romania; catinean1972@yahoo.com; 2 University of Medicine and Pharmacy “Grigore T. Popa” Iasi, 700115 Iasi, Romania; 3 Department of Gastroenterology, “Octavian Fodor” Regional Institute of Gastroenterology and Hepatology, 400158 Cluj-Napoca, Romania; mezei_anita@yahoo.com; 4 Emergency Clinical County Hospital Cluj, 400006 Cluj-Napoca, Romania; botanemil@gmail.com; 5 Pharmacology, Toxicology and Clinical Pharmacology Department, Iuliu Hatieganu University of Medicine and Pharmacy, 400012 Cluj-Napoca, Romania; andrei.mitre97@gmail.com (A.-O.M.); maria.neag@umfcluj.ro (M.-A.N.); 6 Department of Surgery, Emergency Clinical County Hospital Cluj, 400006 Cluj-Napoca, Romania; motocu@gmail.com; 7 Department of Surgery, “Octavian Fodor” Regional Institute of Gastroenterology and Hepatology, 400158 Cluj-Napoca, Romania; graurf@yahoo.com; 8 Department of Surgical and Gynecological Oncology, “Ion Chiricuta” Oncologic Institute, 400010 Cluj-Napoca, Romania; tudor.eniu@umfcluj.ro

**Keywords:** rectal cancer, endoscopic ultrasound, tumor staging

## Abstract

Endorectal ultrasound applications in the evaluation of rectal tumors could be a useful tool in achieving proper staging of rectal cancer. The purpose of this study was to compare the efficacy of rectal tumor staging by flexible endoscopic ultrasound (EUS) with real-time elastography (RTE) using the gold standard post-surgery histological analysis of the resected tissue as the control. The second aim of our research was to establish cutoff values for the EUS-RTE strain ratio corresponding to stages by independently comparing the stiffness values obtained with histology and EUS-RTE staging in order to minimize observation bias. We evaluated the records of 130 patients with a rectal tumor confirmed by biopsy. EUS was used in 70 patients, EUS-RTE—in the other 60. We found no statistically significant differences in staging accuracy when comparing EUS to EUS-RTE. Through a correspondence method between staging assessment and the EUS-RTE stain ratio, we identified cutoff intervals for T2, T3, and T4 staging that were nonoverlapping and proved to be statistically significant in terms of EUS-RTE values (significantly different ascending values from one interval to the other). We found that EUS-RTE offers slightly better, although not statistically significant sensitivity and specificity for T and N stage predictions compared to 2D EUS. Our results showed that EUS-RTE offers slightly higher sensitivity and specificity compared to EUS. Reliable cutoff intervals were found for strain rate elastography, previously available only for shear wave elastography (SWE) which is currently unavailable on any EUS system. Thus, these commonly available EUS-RTE systems can serve as a complementary tool in the staging of rectal tumors.

## 1. Introduction

Colorectal cancer is one of the most common types of cancer in both men and women and has a high rate of morbidity and mortality in developed countries [1]. Treatment is mainly determined by the type and stage of the tumor, so screening and tumor staging are very important tools for prevention and therapy [2]. Nowadays, the imaging tools used for identifying and staging rectal tumors include endorectal ultrasound (EUS), magnetic resonance imaging (MRI), computed tomography (CT), and positron emission tomography (PET) [3]. All the available ultrasound elastography methods use ultrasound to measure the internal tissue shear deformations resulting from the applied force. Strain elastography (SE), particularly real-time elastography (RTE), is the most widely implemented elastography method in commercial ultrasonography systems. It is a qualitative technique using static elastography as the applied force varies slowly and the recorded images suggest the quality of the tissue and its properties. SE depends on the quality of B-mode images. The strain image is converted to numerical values using the color scale (0 being blue and 255 being red). Sometimes, stiffness and fibrosis are inversely correlated with the mean strain values [4,5].

Elastography may improve the staging of rectal cancer and differentiate adenoma from adenocarcinoma when compared to ERUS alone or to MR imaging [5]. Diagnosis and staging of rectal cancer are confirmed by histological examination. Our goal was to determine which is more accurate, EUS or EUS-RTE, in evaluating rectal tumors as reflected in pathology.

Managing colorectal cancer is determined by the tumor type and stage [1,2,3]. As tumors may be characterized by different stiffness levels, strain elastography (SE) may be used in the endosonographic evaluation and staging of cancers [4,5].

The aim of this retrospective study was, first, to evaluate the performance of staging of rectal cancer using flexible EUS applications compared to postoperative histology findings. Afterwards, using an EUS scope with SE capabilities, we aimed to determine whether elastography brings more accuracy when compared with histology. Subsequently, we searched for the intervals of the strain rate (B/A) around which the transition from one stage to another is recorded.

## 2. Materials and Methods

From January 2009 to June 2019, data from 130 patients (68 men and 62 women) with the mean age of 62.6 years (age range: 34–82 years) with histologically confirmed invasive rectal adenocarcinoma were collected for this retrospective single-center study, Cluj-Napoca, Romania. The data were collected from our center’s electronic database. Only the patients that presented data for all the followed parameters were included in the study. All the patients’ analysis reports and images were reevaluated by our team. The patients with a history of rectal surgery and those with metastatic disease were excluded. Among the included patients, 62 underwent neoadjuvant radiotherapy and 13 were managed by chemoradiation prior to study enrolment and EUS evaluation. All the patients were evaluated prior and after neoadjuvant therapy and those who exhibited a significant EUS downstage of the tumor after neoadjuvant radiotherapy or chemoradiation were excluded from this study. All the patients signed the informed written consent form prior to enrolment.

The patients were divided into two sequential groups: seventy patients formed group 1 (January 2009–June 2016) and sixty patients formed group two (June 2016–June 2019). Group 1 was evaluated for tumor staging by Olympus UM-130 (7–12 MHz) EUS and EU-30 M using a mechanical radial scope while group 2 was evaluated by Pentax 3870 UTK (5–10 MHz) EUS and a Hitachi Avius ultrasound machine equipped with the real-time elastography (RTE) software using an electronic radial endoscope. The elastography method results in a color-coded strain map which is superimposed on the B-mode image in real time. Patient number, age, gender, and histologically determined T stage characteristics are displayed in Table 1.

Hard tissue (minor strain) is indicated with dark blue, whereas soft tissue (distinct strain) is indicated with red. As indicated by the color bar in the EUS-RTE images, green and yellow represent intermediate tissue elasticity. A sufficient quantity of reference tissue surrounding the lesion of interest was included in the analysis. The lesion of interest covered 80 to 100% of the surface.

The elastography strain ratio (B/A) was measured for each lesion. The ratio was calculated automatically by a US device after sampling two regions of interest (ROI). One ROI was placed in a focal lesion (A) not more than 0.5 cm from the transducer and the reference ROI was placed adjacent or contralateral to the normal rectal wall structure (B). Three B/A reports were measured, and then the mean value was recorded.

The evaluation of rectal tumors was performed according to the tumor–node–metastasis (TNM) classification system. We assessed tumor growth through rectal wall layers. The patients were prepared for rectal EUS with an oral purgative solution (PEG-3350). All the examinations were performed by the same operator one week prior to the scheduled surgical intervention.

### 2.1. Histopathologic Examination

All the patients underwent surgery: 55 patients—immediately after endoscopy, 62 patients—after short-term radiotherapy, 13—after chemoradiation. The patients with EUS-documented downstage after neoadjuvant therapy were excluded from the analysis.

The surgical samples were fixed with 10% formalin and embedded in paraffin. The tissue slices were stained with hematoxylin and eosin. The hematoxylin-and-eosin-stained slices were evaluated by an experienced colorectal cancer pathologist. The pathological determination of the T and N stages was used as the reference standard according to the guidelines of the American Joint Committee on Cancer (AJCC).

### 2.2. Statistical Analysis

Statistical analysis was carried out using the IBM SPSS Statistics (version 21) software. Pathological findings after surgery were used as the gold standard. Due to the particularities of the data, the strain ratio being numerical and EUS, EUS-RTE being scores, we decided to apply a nonparametric approach.

However, some Gaussian parametric methods were used for a particular analysis (such as ANOVA or comparison of the means). By testing the normality of both numerical data and scores data through the Kolmogorov–Smirnov test for normal distribution concordance, we proved that stiffness sampling values were normally distributed (K–S *p* = 0.128), while for staging scores, all the K–S results were, on the contrary, non-normal (*p* < 0.05). From this result, it was possible to use parametric methods for dealing with stiffness values, like multiple Bonferroni comparisons between interval means, and nonparametric methods when dealing with both stiffness and staging values. With regards to the means and standard deviation of staging scores, such calculations were carried out just for establishing the tendencies of staging assessments but were not interpreted as being of medical significance. Fractions of scores do not have medical interpretation, only integer values do.

To identify the concordance between the diagnostic methods, the Kendall’s concordance test was applied. Furthermore, in order to determine if the stage determined by one method (EUS or RTE) corresponded to the same stage determined by the other method (histology), a sensitivity/specificity analysis was carried out and both specificity and sensitivity were determined. The ANOVA test was used for multiple comparisons between the cutoff intervals for testing that they were consecutive. By overlapping the other staging methods over the B/A calculation, the corresponding stiffness intervals were established. These proved to be distinct. Bonferroni pairwise comparisons between stiffness intervals were used for the 0.05 statistical significance and 95% confidence intervals (95% CI) for the center of the intervals. This finding allowed recommending the limits of these intervals as cutoff values for stiffness. The cutoff intervals, 95% confidence intervals (established for the B/A ratio) represent the estimated values for the rigidity values. We reported the statistically significant results for *p* < 0.05 and 95% CI of the cutoff values.

## 3. Results

**Figure 1:**
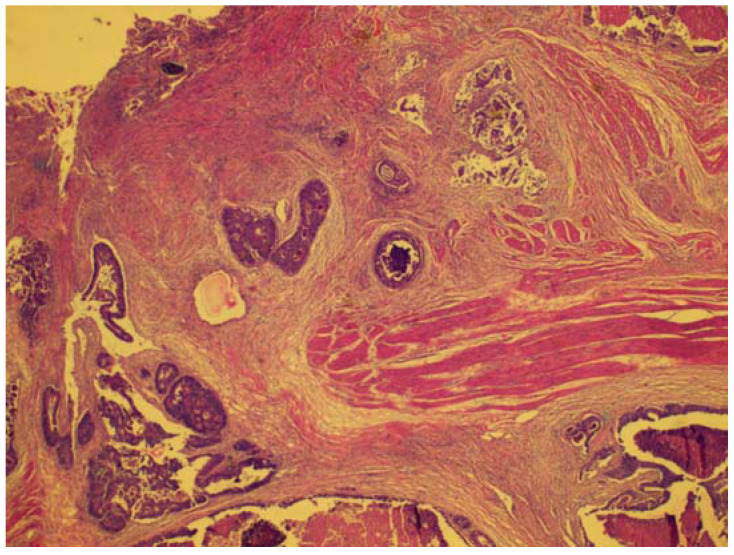
A rectal wall with ulcerated mucosa (top) and tumor islands infiltrating into the muscular propria layer.

The data obtained in both the analyzed cohorts (EUS and EUS-RTE) were compared with the histological staging considered the gold standard in the diagnosis of rectal cancer extension. The comparison between the T stage obtained by EUS (EUS_T) and EUS-RTE (EUS-RTE_T) and that established by histological analysis (HIS_T) showed that there were no statistically significant differences neither in the first group (Kendall’s concordance coefficient of 0.032, with *p* = 0.134) (the mode, the most frequent stage, for EUS_T was 3 as well as for HIS_T) nor in the second group (Kendall’s concordance coefficient of 0.086, with *p* = 0.166) (the mode of EUS-RTE_T was 3 as well as for HIS_T). These tests showed that the distribution of values determined both by EUS and EUS-RTE was comparable to that determined by histological analysis (Figure 1). The results obtained for T staging with EUS and EUS-RTE are summarized in Table 2.

The same comparison was made for the data obtained for the N stage. The two diagnostic evaluations, EUS and histological analysis for N staging, indicated no statistically significant differences (Kendall’s concordance coefficient of 0.058 and *p* = 0.058) (the mode, the most frequent stage, of EUS_N was 0 as well as for HIS_N). The comparison between the N stage obtained by EUS-RTE and histological analysis showed that there were no significant differences (Kendall’s concordance coefficient of 0.054 and *p* = 0.071) (the mode of EUS-RTE was 0 as well as for the HIS_N). The results obtained for the N stage with EUS and EUS-RTE, respectively, are summarized in Table 3.

**Figure 2:**
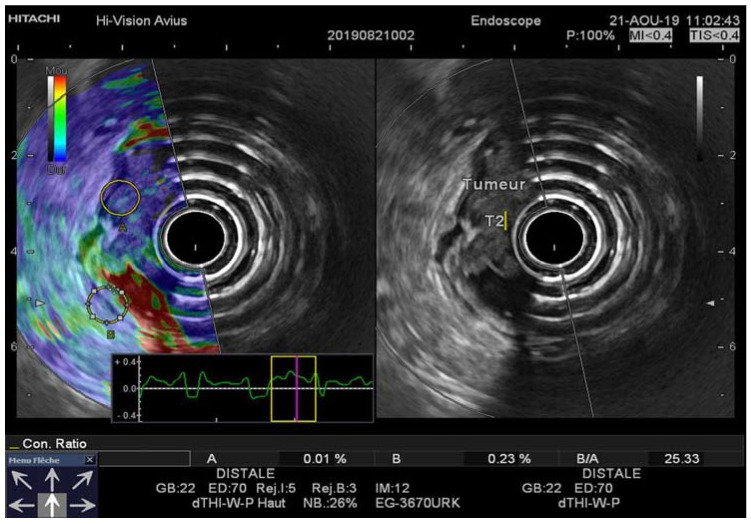
(**A**) EUS-RTE aspect in a 59-year-old male with pT2 rectal cancer. The tumor was classified as T2 by using the strain ratio (B/A); (**B**) EUS aspect of the same tumor confirmed by histopathologic examination after surgical resection. RTE, real-time elastography; EUS, flexible endorectal ultrasound.

The subsequent goal of our study was to evaluate the values around which the transition from one stage to another could be observed. Therefore, the T stage cutoff values obtained by performing EUS-RTE and histological analysis were taken as the benchmark. Staging performed by EUS-RTE or histological analysis for the T stage was used as the grouping variable of the B/A stiffness values (Figure 2).

The results are shown in Table 4. A single T4 stage patient was identified by histological analysis, which is why this was not included in the analysis.

The ANOVA test was applied to compare the T stage results obtained by EUS-RTE and histological analysis, respectively, in order to show that the intervals were independent. The results obtained in both cases (EUS-RTE and histological analysis) showed that the three intervals (T2, T3, and T4) did not overlap and there were statistically significant differences (*p* = 0.0001 and *p* = 0.001, respectively).

**Figure 3:**
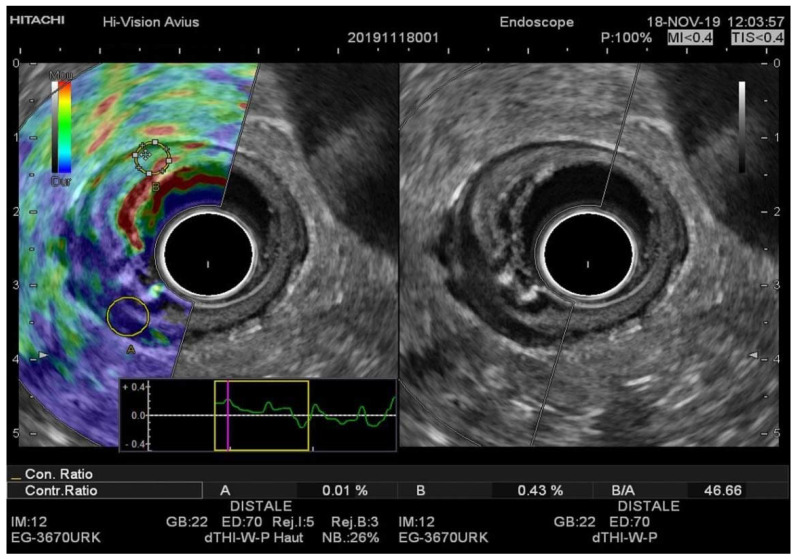
(**A**) EUS-RTE aspect in a 67-year-old female with pT3 rectal cancer. (**B**) EUS aspect of the same tumor. The tumor was classified as T3 with the cutoff value strain ratio (B/A) of 46.66. T3 was confirmed by histopathologic examination after surgical resection. RTE, real-time elastography.

**Figure 4:**
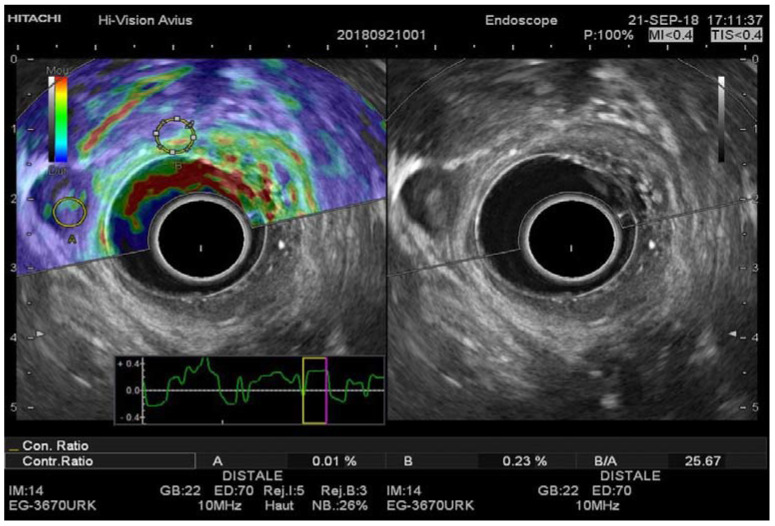
Images of a 60-year-old man with pT2N1 rectal cancer. Regions of interest for the EUS-RTE measurements were placed on a suspicious lymph node (**A**) and the normal portion of the rectal wall (**B**), superimposed on the B-mode image. RTE, real-time elastography.

Based on our results, we determined possible cutoff intervals for T staging established by EUS-RTE (Figure 3). Subsequently, we proved better specificity of the EUS-RTE method for N staging compared to EUS and also better sensitivity for N0 and N1, but not for N2 (Figure 4).

## 4. Discussion

Accurate staging of rectal cancer influences treatment decision, morbidity, and mortality [4,5,6,7]. Waage et al. reported that strain ratio measurements in strain elastography were significantly different between adenomas and adenocarcinomas. Moreover, the use of strain ratio measurements to discriminate benign lesions from malignant ones offers high sensitivity (82.0%), specificity (86.0%), and accuracy (84.0%), meaning that strain elastography can provide added value when used in conjunction with endorectal US [8,9]. These images, however, do not directly quantify the elasticity of the tumor. Our study differs from Waage’s in the fact that we included patients with invasive adenocarcinoma and used EUS for rectal cancer staging. T staging was underestimated in six cases with T2 instead of T3 and overestimated in 18 cases (11 cases with T3 instead of T2; seven cases with T4 instead of T2 or T3). Using SR-RTE for rectal cancer, the T stage was underestimated in four cases (T2 instead of T3), which may have resulted in the loss of an opportunity for neoadjuvant therapy or extensive surgery and preventing local recurrence.

T staging was overestimated in five cases (T3 instead of T2), which could have resulted in unnecessary therapy and, subsequently, associated morbidity. Thus, the results showed slightly better sensitivity and specificity for SR-RTE compared to EUS, but with no significant differences (Table 2 and Table 3). One staging challenge of endorectal US is differentiation between stages T2 and T3. A reduced accuracy is noted for stage T2 tumors. Patients with stage T2 tumors are easily over-staged as stage T3 due to adjacent inflammation, locally retained secretions, or desmoplastic reactions. EUS is limited in identifying inflammation, fibrosis, and lymph nodes. These reactive changes are also hypoechoic with an irregular outer layer of the rectal wall at endorectal US similar to the transmural tumor extension.

Thus, misinterpretation of tumor depth can lead to higher T staging; underestimation of tumor infiltration depth could be a consequence of the low ability to detect certain microinvasions or may be caused by microscopic tumor invasion into the perirectal fat. Endoluminal air and rectal fecal residues may also affect appreciation of the depth of tumor invasion [10].

In our cohort, a significant variation in sensitivity between the T2/T3 and T4 stages was observed. Therefore, both EUS and EUS-RTE are a good choice for diagnosis, especially in the case of mild and moderate rectal malignancies. For the staging of advanced lesions, other methods with higher sensitivity such as MRI are recommended [11]. The data and images obtained by SR-RTE can be considered complementary information obtained by conventional EUS [12]. In accordance with previous reports, we found that the stiffness of carcinomas was higher than that of adenomas [13]. Consequently, the stiffness of tumors rose gradually with depth of infiltration.

The mean values of such intervals reported with standard deviations are presented above in Table 3. Since the intervals for T2, T3, and T4 show statistically significant differences, we can conclude that stiffness can be divided into intervals. These intervals could be used for T staging using this method, but the optimal cutoff values should be further verified in more extensive cohorts.

In the pilot study of Li-Da Chen, individual stiffness values were obtained to distinguish adenomas from adenocarcinomas. They concluded that tumor stiffness increased gradually with the increase of the depth of infiltration [14].

Nevertheless, the so far determined cutoff values are inhomogeneous and scarcely comparable, as the authors used either absolute strain values or the strain ratio to compare the neoplastic lesion with various anatomic structures nearby. By using the feature of tumor stiffness for EUS-RTE, the accuracy of preoperative staging for rectal tumors was superposable with histological results in this pilot study.

In addition to T staging, N staging (to determine the extent of the nodal disease) is also important in therapy management. For the N stage, only mesorectal and internal iliac nodes (regional nodes) are considered within the TNM system. Other nodes involved are considered metastases [15]. Although the characterization of pathological lymph nodes does not include their elasticity, in practice, during elastography, they appear stiffer than the perirectal fat [16].

A meta-analysis performed by Puli et al. demonstrated that EUS is an important method for excluding nodal invasion rather than confirming the presence of nodal-positive disease [17].

According to the comparison of EUS with EUS-RTE, the latter requires more technical skills as it could be affected by the positioning of the probe against the tumor, removal of the air- or water-filling balloon, peristaltic movements of the wall of the intestine [18]. Furthermore, it is important to note that the staging of rectal cancer is highly dependent on the skills of the operator. The examination must be carried out by an experienced operator [19]. However, some limitations of this study should be noted. First, the quality of the EUS scope may have significantly improved between the two time periods compared. Second, technical challenges may arise during the recording of elastograms caused by endoluminal air and rectal fecal residues. Furthermore, given the study design, the interobserver variability of the diagnosis was not assessed. Finally, it was not possible to evaluate by EUS neither the mesenteric lymph nodes nor the extension of the posterior tumor beyond the endopelvic fascia in the advanced colorectal cancer highlighted.

## 5. Conclusions

Our study showed that EUS-RTE offers slightly higher sensitivity and specificity compared to EUS. EUS-RTE efficiency in predicting the T or N stage of rectal cancer is just slightly higher than that of EUS. The results are supported by a strong correlation between the results obtained by EUS-RTE with the histologically obtained T and N stages. Thus, the evaluation of rectal neoplasms by EUS-RTE may improve the staging of rectal adenocarcinomas compared with EUS.

Moreover, EUS-RTE is a method with major advantages: it is commercially available on the majority of ultrasound systems, cutoff values for each T stage reduce observer subjectivity, and patient compliance is high for flexible EUS probes. However, further studies with larger cohorts should be performed in order to evaluate the value of combined EUS and EUS-RTE assessment as an integrated part of the pretreatment algorithm for rectal cancer and to evaluate its impact on planning multimodal therapy.

## Figures and Tables

**Table 1:** Patients’ characteristics.

	Overall	EUS Group	EUS-RTE Group
Number of patients	130	70	60
Age (years), mean (±SD)	62.44 (±9.67)	59.78 (±9.04)	65.5 (±9.52)
Male gender, *n* (%)	68 (52.3%)	52 (74.28%)	16 (27%)
T1 stage, *n* (%)	9 (6.92%)	9 (12.86%)	0
T2 stage, *n* (%)	36 (27.69%)	20 (28.57%)	16 (26.67%)
T3 stage, *n* (%)	76 (58.46%)	34 (48.57%)	42 (70%)
T4 stage, *n* (%)	3 (2.31%)	1 (1.43%)	2 (3.33%)

Abbreviations: EUS, endorectal ultrasound; RTE, real-time elastography.

**Table 2:** Specificity and sensitivity, EUS_T and EUS-RTE_T.

EUS, T Stage	Specificity	Sensitivity	EUS-RTE, T Stage	Specificity	Sensitivity
T2	60%	71.19%	T2	78.14%	78.14%
T3	58.12%	71.27%	T3	68%	85.11%
T4	72.11%	12.5%	T4	83.13%	33.13%

RTE, real-time elastography; EUS, flexible endorectal ultrasound.

**Table 3:** Specificity and sensitivity, EUS_N and EUS-RTE_N.

EUS, N Stage	Specificity	Sensitivity	EUS-RTE, N Stage	Specificity	Sensitivity
N0	22.15%	97.12%	N0	63.3%	86.7%
N1	95%	43.13%	N1	96.29%	80%
N2	73.14%	75%	N2	84%	30%

RTE, real-time elastography; EUS, flexible endorectal ultrasound.

**Table 4:** Cutoff stiffness for the T stage evaluated by EUS-RTE and histological analysis.

	EUS-RTE_T			HIS_T	
Stage	T2	T3	T4	T2	T3
Cutoff intervals with 95% CI	22–39 30.95 ± 4.05	41–53 47.23 ± 2.94	40–83 62 ± 8.42	28–41 35.31 ± 2.98	38–50 44.57 ± 3.08

RTE, real-time elastography; EUS, flexible endorectal ultrasound; HIS, histological analysis.

## Data Availability

Data available on request due to restrictions e.g., privacy or ethical, The data presented in this study are available on request from the corresponding author. The data are not publicly available due to the fact that it is contained within personal records of patients retrospectively analyzed.
